# Genome Sequence of *Burkholderia pseudomallei* NCTC 13392

**DOI:** 10.1128/genomeA.00183-13

**Published:** 2013-05-23

**Authors:** Jason W. Sahl, Joshua K. Stone, H. Carl Gelhaus, Richard L. Warren, Caroline J. Cruttwell, Simon G. Funnell, Paul Keim, Apichai Tuanyok

**Affiliations:** Northern Arizona University, Center for Microbial Genetics and Genomics, Flagstaff, Arizona, USAa; Translational Genomics Research Institute, Flagstaff, Arizona, USAb; Battelle Biomedical Research Center (BBRC), Columbus, Ohio, USAc; Tunnell Consulting, Bethesda, Maryland, USAd; Health Protection Agency, Porton Down, Salisbury, United Kingdome

## Abstract

Here, we describe the draft genome sequence of *Burkholderia pseudomallei* NCTC 13392. This isolate has been distributed as K96243, but distinct genomic differences have been identified. The genomic sequence of this isolate will provide the genomic context for previously conducted functional studies.

## GENOME ANNOUNCEMENT

*Burkholderia pseudomallei* is the causative agent of the potentially fatal disease melioidosis (1). Melioidosis is endemic to Southeast Asia and northern Australia, where *B. pseudomallei* is found in wet soil (2). *B. pseudomallei* is a phylogenetically diverse pathogen, as revealed by whole-genome sequence analysis (3).

A recent study demonstrated that *B. pseudomallei* NCTC 13392 (cited at the time of the study by the United Kingdom National Collection of Type Cultures as strain K96243), provided by the Health Protection Agency (HPA) in the United Kingdom, was shown to cause lethal inhalational melioidosis in a marmoset model (4). We sequenced the isolate used in that study and compared its genome to that of the published version of K96243 (5). Based on a comparison of single nucleotide polymorphisms (SNPs) and phylogenetic position, we determined that NCTC 13392 is substantially different from K96243. Subsequent investigation identified that although NCTC 13392 was received from the same laboratory in Thailand at the same time as K96243, it was unexpectedly a different strain; the authors of the marmoset study have since published an erratum to describe this discrepancy (6). Here, we describe the genome to provide the context for the functional results obtained from the marmoset model study.

Genomic DNA was isolated with Wizard Genomic DNA purification kit (Promega, Inc.). The genomic sequence was generated on the Illumina GA IIx platform, using both 300-bp and 1,000-bp insert sizes. A comparative assembly was performed with AMOScmp (7) with the 1,000-bp insert sequences, using K96243 as the reference sequence, based on preliminary phylogenetic analyses. The AMOS assembly was then processed with the PAGIT pipeline (8) to improve the contiguity of the assembly. A *de novo* assembly was also performed with Velvet (9) in conjunction with the VelvetOptimiser (http://bioinformatics.net.au/software.velvetoptimiser.shtml). The reference-based assembly and the *de novo* assembly were then aligned with Mugsy (10). Regions unique to the *de novo* assembly were parsed from the alignment and concatenated to the comparative assembly. Errors were corrected in the final assembly with iCORN (11). The raw reads were also mapped to the final assembly with the Burrows-Wheeler Aligner (BWA) (12), and SNPs were called with the Genome Analysis Toolkit (GATK) (13).

The final assembly consisted of 48 contigs, with an assembled genome length of 7.16 Mbp and an N_50_ of ~418 Kbp. The average coverage across the genome was 140× (1,000-bp library). An *in silico* multilocus sequence type (MLST) analysis classified NCTC 13392 as sequence type 23 (ST23), with a single nucleotide difference to K96243 in the *gltB* gene. There are 7 ST23 isolates in the MLST database (http://bpseudomallei.mlst.net/), all of which were isolated in Thailand. An SNP analysis of raw reads against the finished genome of K96243 identified 13,256 SNPs, including 3,050 nonsynonymous SNPs. In addition, ~121 Kbp of sequence data was identified in NCTC 13392 that was absent from K96243; genes from these regions were associated with bacteriophages in previously sequenced *B. pseudomallei* genomes. This analysis demonstrates that the genome of NCTC 13392 is distinct from that of K96243 and that genomic elements from NCTC 13392 might be used to correlate with the results from animal model studies.

### Nucleotide sequence accession numbers.

This Whole-Genome Shotgun project has been deposited at DDBJ/EMBL/GenBank under the accession no. AOUG00000000. The version described in this paper is the first version, accession no. AOUG01000000.
